# Comparison of Physical Activity Patterns among Three Major Chronic Respiratory Diseases

**DOI:** 10.3390/jcm12216832

**Published:** 2023-10-29

**Authors:** Leandro Cruz Mantoani, Karina Couto Furlanetto, Carlos Augusto Camillo, Joice Mara de Oliveira, Cláudia Polastri, Lorena Paltanin Schneider, Camile Ludovico Zamboti, Nidia Aparecida Hernandes, Fabio Pitta

**Affiliations:** 1 Laboratory of Research in Respiratory Physiotherapy (LFIP), Department of Physiotherapy, Londrina State University (UEL), Londrina 86047-970, Brazil; ka_furlanetto@hotmail.com (K.C.F.); carlos.a.camillo@uel.br (C.A.C.); joice.mara.oli@gmail.com (J.M.d.O.); polastriclaudinha@gmail.com (C.P.); lorena.pschneider@gmail.com (L.P.S.); camileludovico@gmail.com (C.L.Z.); nyhernandes@gmail.com (N.A.H.); 2 Department of Physiotherapy, Faculty of Science and Technology, São Paulo State University (UNESP), Presidente Prudente 19060-080, Brazil; 3 Graduate Associated Program in Rehabilitation Sciences, University Pitagoras UNOPAR / UEL, Londrina 86041-140, Brazil

**Keywords:** physical activity, chronic respiratory disease, COPD, asthma, idiopathic pulmonary fibrosis

## Abstract

Although the level of physical activity in daily life (PADL) plays a vital role concerning the health of subjects with chronic lung diseases, it remains uncertain how PADL patterns compare among different conditions. This study’s objective was to compare the PADL levels of subjects with COPD, asthma and idiopathic pulmonary fibrosis (IPF); and to investigate PADL behaviour in different diseases’ severity. Stable subjects who had not undergone pulmonary rehabilitation in the previous year were included. Subjects were divided into two subgroups according to disease severity: mild/moderate and severe/very severe. The primary outcome was time spent in moderate-to-vigorous physical activities (MVPA) (Actigraph GT3x) measured during one week over 12 h/day; other assessments included pulmonary function, peripheral muscle strength and exercise capacity. Comparisons among subgroups were corrected for age, BMI and sex. The analysis involved 119 subjects (47 asthma, 48 COPD and 24 IPF). Subjects with asthma had higher PADL levels than those with COPD and IPF (MVPA 18(14–22) vs. 8(4–12) vs. 7(1–12) min/day, respectively; *p* ancova = 0.002). Subjects with severe/very severe IPF had the lowest PADL level among all subgroups. Adult subjects with asthma have higher PADL levels than those with COPD and IPF, whereas patients with severe and very severe IPF are the most physically inactive subjects.

## 1. Introduction

Physical activity in daily life (PADL) plays a key role in maintaining overall well-being and health in the general population [1,2]. Its significance becomes even more noticeable when considering subjects with chronic respiratory conditions such as chronic obstructive pulmonary disease (COPD), asthma and idiopathic pulmonary fibrosis (IPF), since inactivity is associated with poor outcomes in these populations [3,4,5,6].

Subjects with chronic respiratory diseases are characterized by dyspnoea sensation and exercise intolerance [7,8,9], which lead to a physically inactive behaviour in daily life [6,10,11]. This inactivity can reduce muscle strength in the lower limbs, which might induce physical deconditioning, further enhancing the dyspnoea sensation. Consequently, these subjects can enter in a vicious cycle of inactivity, dyspnoea and deconditioning, which is quite characteristic of some chronic respiratory conditions [12].

Although the level of PADL has an important role concerning the health of subjects with chronic respiratory diseases, it remains unclear how PADL patterns compare among different conditions. Understanding the specificities of PADL levels among subjects with COPD, asthma and IPF may be vital for targeting tailored interventions which aim to improve their functionality, overall quality of life, exacerbation rates and even mortality.

These three chronic respiratory conditions, while different in their physiopathology, aetiology, disease progression and survival rates, share some common features of impaired lung function, dyspnoea sensation, decreased exercise capacity and reduced muscle strength [7,8,9,12,13]. Still, the patterns of PADL levels of these populations can differ significantly, meaning that for each subgroup, unique challenges and opportunities to engage in (or maintain) an active lifestyle might be needed. By contrasting the PADL patterns of COPD, asthma and IPF subjects, we can provide valuable insights into tailored approaches for managing and improving the inactivity in these chronic respiratory conditions.

COPD, asthma and IPF are among the main chronic respiratory conditions found worldwide [7,8,9], affecting millions of people on the planet. They have important implications for healthcare providers due to their hospitalization and mortality rates, as well as their cost for health services, while being some of the most investigated pulmonary disorders by the scientific community. Although individuals with COPD, asthma and IPF have some similarities in terms of clinical parameters, their PA levels (and consequently their PA behaviour) are not necessarily the same. Subjects with different diseases may have a completely different PADL due to the nature of their conditions. Also, the severity of the disease might play a role in PA levels when comparing subjects from different populations. Therefore, it seems plausible to investigate and to compare these (sub)populations according to their PA levels.

Due to the different characteristics of the diseases, we hypothesized that adult subjects with asthma would be more active in daily life than those with COPD or IPF. Therefore, the aims of the present study were to compare the PADL levels of three major chronic respiratory conditions: asthma, COPD and IPF; and to analyse the PADL behaviour in the diseases’ subgroups according to severity (mild/moderate and severe/very severe).

## 2. Materials and Methods

The present study is a secondary analysis of baseline data collected for three previous unrelated research projects developed in the Laboratory of Research in Respiratory Physiotherapy (State University of Londrina, Brazil) involving the three different populations (COPD, asthma and IPF). Two projects received approval by the research ethics committee of the State University of Londrina (Brazil) under the numbers 1,730,247 (COPD) and 5,697,474 (IPF), and another by the research ethics committee of the University Pitagoras UNOPAR (Brazil) under the number 3,060,314 (asthma). All participants signed a written informed consent before partaking in the studies.

The project involving individuals with asthma assessed a number of different outcomes at baseline. These outcomes included lung function, PADL, functional tests, exercise capacity and health-related quality of life, among others. The project involving individuals with COPD was a study aiming to investigate the effects of an exercise programme in different outcomes in COPD, especially PADL. The data coming from the project on IPF was the baseline assessment of a longitudinal cohort study aiming to investigate clinical endpoints and prognosis in interstitial lung diseases. Even though there were some longitudinal data involved in the projects, all the data analysed in the present study were collected at the baseline moment only [14,15,16,17]. None of the mentioned projects had any impact on subjects’ PADL at the baseline time; therefore, the present results were not influenced by the fact that a secondary analysis of partial data collected in other projects was performed.

Participants were referred by medical doctors or invited by letters/advertisements to join each respective project. The list of specific inclusion and exclusion criteria for each study can be found elsewhere [14,15,16,17]. Subjects were consecutively assessed/included in each of these three previous projects, and included currently in the present analysis if they fulfilled the following criteria: they had to be diagnosed with COPD, asthma (adults) or IPF according to international criteria [7,8,18,19]; they should have been clinically stable for at least four weeks prior to their baseline assessment (i.e., no exacerbation of the disease); they could not have participated in pulmonary rehabilitation programmes in the previous year; they could not have more than one of the respiratory diseases concomitantly; they could not have any severe orthopaedic, neurological, cognitive and/or psychiatric impairment hindering mobility.

For the present analysis, subjects were divided into severity subgroups according to international criteria. For subjects with COPD, we used the GOLD criteria I–IV [7], grouping together subjects GOLD I + II (mild + moderate) and GOLD III + IV (severe + very severe). As for subjects with asthma, we used the GINA steps for medication that range from 15, where 1–3 are the mild to moderate asthmatic subjects, and 4–5 are the severe and very severe ones [8]. Since there is no global consensus to stratify subjects with IPF according to the disease severity, we based this classification on a study by Antoniou et al. [19] which used the FVC in % of predicted values to classify severity, so that subjects with an FVC below 50% of predicted values were classified as severe-to-very-severe IPF, and those above this value as mild-to-moderate disease. These values have been used by other studies, especially because they relate to severe physiological impairments [20] and also the risk for disease progression in this population [21].

A comprehensive set of assessments was performed in each participant, including the following: PADL (monitored over the course of one week by a physical activity [PA] monitor), pulmonary function, exercise capacity and peripheral muscle strength (please see below in detail). General information on medical history and health status, including demographic details, COPD/asthma/IPF history and exacerbation records, were also collected. Other assessments were performed in each specific project; however, they are unrelated to the present analysis.

The primary outcome of the present analysis was the level of PADL. The assessment of PADL as an outcome was performed in all three samples by the Actigraph GT3x accelerometer (or PA monitor) (Actigraph^®^, Pensacola, FL, USA), which is well-established and validated for use in chronic respiratory diseases [22,23]. This device offers multiple variables to evaluate PA, including the number of steps/day, number of metabolic equivalent task rates (METs), energy expenditure in Kcal and time spent in different PA intensities (e.g., sedentary, light, moderate, vigorous and very-vigorous, please see below). Participants wore the PA monitor throughout their waking hours over the course of seven consecutive days. To ensure the accuracy of data collection, PA levels were calculated as the average of measurements collected on a minimum of four valid weekdays [24], with each valid day requiring a minimum usage length of twelve hours [24]. Therefore, individuals with less than 4 valid weekdays of less than 12 h of monitorization were not included in the analysis. For the purpose of standardization and data compilation, we analysed the PA levels in all subjects from 07:00 am to 07:00 pm, dividing the morning from 07:00 am to 12:59 pm, and the afternoon from 01:00 pm to 06:59 pm. We used the total time spent/day in moderate-to-vigorous PA (MVPA) (i.e., intensity > 3 METs, expressed in minutes/day) as the main variable of the study. However, we have also analysed PADL data using the following variables: number of steps/day, time spent/day in sedentary activities (i.e., intensity < 1.5 METs, expressed in minutes/day) and time spent/day in light activities (i.e., intensity ranging from 1.5 to 3 METs, also expressed in minutes/day).

The accelerometer counts were translated into minutes of physical activity by using the Freedson bout parameters [25]. We used a minimum length of 10 min bouts to consider both Freedson’s bout parameters and the sedentary bout parameters. The minimum count value for sedentary bout parameters per minute was 0, and the maximum count value was 99 counts per minute. As for the cut point values for each type of activity, we used the following ranges: 0–99 for sedentary activities; 100–1951 for light activities; 1952–5724 for moderate activity; 5725–9498 for vigorous activity; and 9499 and above for very vigorous physical activity. Therefore, the MVPA minimum count was considered to be 1952. We have chosen the Freedson bout parameters since they have been widely used in clinical research, including adult subjects with chronic respiratory diseases [26].

The secondary outcomes of the study were pulmonary function, exercise capacity and peripheral muscle strength. Pulmonary function was evaluated by spirometry and whole-body plethysmography (Vmax^®^ Carefusion, Yorba Linda, CA, USA). These evaluations were conducted following the protocols outlined in the American Thoracic Society/European Respiratory Society statement [27,28], which involved determining parameters such as the forced expiratory volume in the first second (FEV_1_), forced vital capacity (FVC), the FEV_1_/FVC ratio and the maximal voluntary ventilation (MVV). All the measurements were taken pre- and post-administration of bronchodilator medication to ensure accuracy, with post-bronchodilator values used for the analysis. Reference values by Pereira et al. [29] were used. Exercise capacity was assessed by the 6-min walking test (6MWT). According to the recommended international criteria [30], the test was performed on a 30 m flat corridor, where participants were instructed to cover the longest walking distance possible during 6 min. The test was performed twice, with a 30 min interval between them, and the highest distance covered in the test was utilised for analysis. Specific reference values for the Brazilian population were used [31]. Peripheral muscle strength was measured by the maximum voluntary isometric contraction of the quadriceps femoris (QF), performed on a multi-gym device attached to a strain-gauge system (EMG System^®^, São José dos Campos, SP, Brazil). Patients performed the test seated with a 90-degree hip and knee flexion, with their arms crossed over their chest. The dominant leg was assessed and the highest possible value of maximal QF contraction (during 6 s) was used for analysis [32].

Statistical analysis was carried out using the Statistical Package for the Social Science—SPSS version 21—(IBM^®^, New York, NY, USA) and GraphPad Prism 6.0 (GraphPad Software^®^, Boston, MA, USA). The Kolmogorov–Smirnov test was used to analyse the normality in data distribution. Depending on that, data were described as mean (95% confidence interval—CI) or median [interquartile range 25–75%]. The Kruskal–Wallis test (with Dunn’s post hoc test) was used to compare the characteristics of the included subjects in each group (asthma vs. COPD vs. IPF). The analysis of co-variance (ANCOVA) with Sidak’s correction was performed for the comparison of outcomes in all groups of chronic respiratory diseases. No baseline pairing among the samples was conducted; however, the analysis was corrected for age, body mass index (BMI) and sex. To perform this analysis, first a one-way ANOVA was performed to guarantee that the independent variable did not have effects over the covariable (confounders). Then, the homogeneity of the regression parameters was tested within the groups of independent variables. Finally, a complete factorial model was used to compare the main effects, adjusting the confidence interval with Sidak’s correction. For the purpose of data analysis, we divided the subjects in each respiratory disease group (COPD, asthmatic and IPF) into two subgroups: mild/moderate and severe/very severe. When comparing these subgroups, we also corrected data analysis for age, BMI and sex. Statistical significance was set at *p* < 0.05.

## 3. Results

A total of 119 subjects who fulfilled the inclusion criteria were analysed (47 subjects with asthma, 48 with COPD and 24 with IPF). As expected, patients with asthma were younger and had a better pulmonary function and exercise capacity than subjects with COPD or IPF. The characteristics of the included subjects can be found in Table 1.

### 3.1. Comparison of Physical Activity Levels among Different Chronic Respiratory Diseases

**Figure 1:**
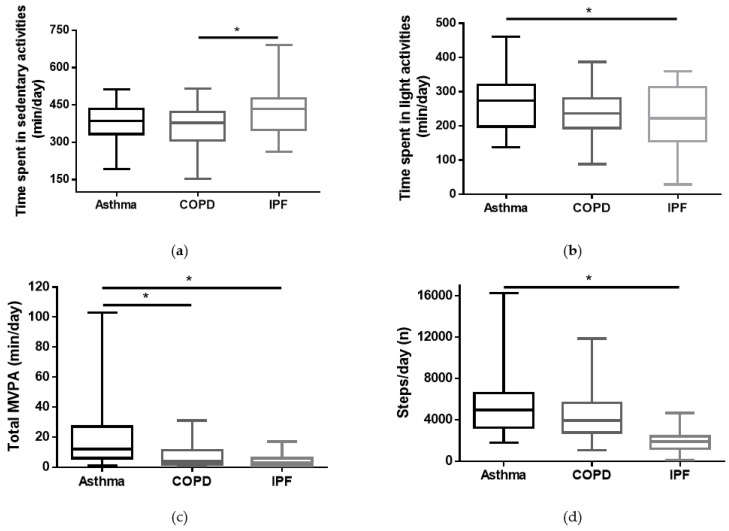
Comparison of PA levels among different diseases corrected for age, BMI and sex: (**a**) time spent/day in sedentary activities (min/day); (**b**) time spent/day in light activities (min/day); (**c**) time spent/day in moderate-to-vigorous physical activity (MVPA) (min/day); (**d**) number of steps/day. * = *p* < 0.05.

As shown in Figure 1, subjects with asthma had higher PA levels than those with COPD or IPF: panel b, time spent/day in light activities 269 (244–293) vs. 232 (209–255) vs. 216 (186–247), respectively, *p* ancova = 0.033; panel c, time spent/day in MVPA 18 (14–22) vs. 8 (4–12) vs. 7 (1–12), respectively, *p* ancova =0.002; panel d, number of steps/day 5543 (4706–6381) vs. 4512 (3729–5295) vs. 3455 (2403–4508), *p* ancova =0.016. Moreover, panel a of Figure 1 shows that subjects with COPD presented a lower time spent/day in sedentary activities when compared to IPF subjects (381 (355–407) vs. 366 (342–390) vs. 428 (396–461), *p* ancova =0.008, (asthma vs. COPD vs. IPF, respectively).

### 3.2. Comparison of Physical Activity Levels among Subjects with Different Severity of Chronic Respiratory Diseases

**Figure 2:**
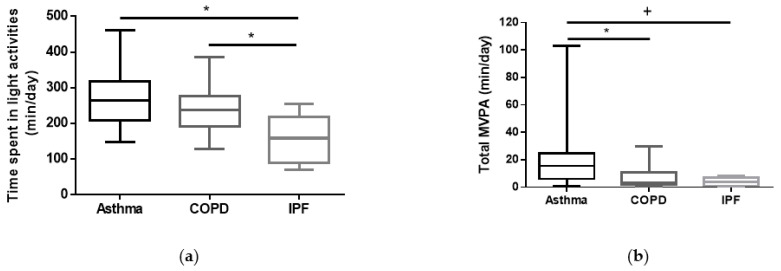
Comparison of time spent/day in light and moderate-to-vigorous physical activities in severe/very severe subjects corrected for age, BMI and sex: (**a**) time spent/day in light activities (min/day); (**b**) time spent/day in moderate-to-vigorous physical activity (min/day). * *p* < 0.05, ^+^ near statistical significance vs. asthma.

When the subgroups divided according to disease severity were analysed, no statistically significant differences in PA levels were observed among mild/moderate subgroups of subjects in the different chronic respiratory conditions (asthma *n* = 12, COPD *n* = 24, and IPF *n* = 18, *p* > 0.05 for all comparisons). However, concerning subjects with severe and very severe disease, Table 2 and Figure 2 show that subjects with severe and very severe IPF had the lowest PA levels among the studied subjects.

## 4. Discussion

The present study sheds light on the distinct PA patterns observed in three major chronic respiratory diseases: COPD, asthma and IPF. Our findings show that subjects with asthma are more physically active in daily life than individuals with COPD or IPF, even when the analysis was corrected for important demographic variables such as age, BMI and sex. Moreover, when comparing patients with different conditions with similar severity of the disease, no statistically significant differences were observed in mild/moderate subjects. However, severe and very severe individuals with IPF were the most inactive patients across all three respiratory conditions. Since lower levels of PA are related to worse outcomes in pulmonary disorders [3,4,5,6], our findings highlight the importance of looking specifically at more severe patients, especially in IPF.

As far as we are aware, this is one of the first studies comparing the PA levels of major chronic respiratory diseases in the scientific literature. A previous study compared daily PA in subjects with COPD, bronchiectasis and severe asthma [33]. The authors found that PA impairment is common across adults with obstructive airway diseases. However, the groups were not clinically matched for factors known to be related to PA, which caused the results to be interpreted with caution. More recently, Breuls and colleagues [34] explored the PA levels of subjects with COPD and compared with subjects with interstitial lung diseases (ILD). The authors matched patients for age, gender, functional exercise capacity and season of assessment. They showed that patients with ILD perform PA at a lower intensity compared to their COPD peers, even though an equal amount of steps/day was achieved throughout the week in both groups. Nevertheless, that study included subjects with different ILD (e.g., IPF and non-IPF) and used an activity monitor that, although validated in COPD [22], has been shown to be not so accurate to measure one of the main outcomes of the study (i.e., number of steps/day) [35]. These limitations do not jeopardize the authors’ findings but may restrict the applicability of their results.

On the other hand, both our findings and methodologies are somewhat unique when compared to these previous studies [33,34]. The present study explored and gathered crucial information not found in both abovementioned studies, such as: (i) PA levels in different respiratory conditions, including pure obstructive and restrictive airway diseases (e.g., only IPF subjects instead of a heterogeneous group of ILD patients); (ii) we performed the analysis correcting for important demographic variables such as age, BMI and sex; (iii) we used a validated PA monitor [22,23] that is accurate to measure daily step counts and time spent in different activities (including sedentary, light and MVPA); and (iv) we have compared the activity levels in varying diseases’ severities. Since daily PA levels, a keystone of health, play a crucial role in managing these conditions, it is likely that these findings add useful information for clinicians and researchers.

The present results reveal notable discrepancies in PA levels among subjects with COPD, asthma and IPF. Subjects with asthma demonstrated significantly higher levels of daily PA compared to those with COPD and IPF (Figure 1). This divergence might be due to differences in disease pathophysiology [7,8,18,19], with asthmatic patients typically being characterized by intermittent symptoms [8] that may allow for a higher level of PADL. In contrast, subjects with COPD and IPF are often related with more debilitating and persistent symptoms/clinical conditions that limit PA, including: worse lung function, dyspnoea sensation and impaired exercise capacity [36]. The observed differences in PA levels among these chronic respiratory diseases hold important implications for clinical practice. Healthcare providers and clinicians should identify the diverse PA needs and abilities of their patients based on their specific diagnosis, and especially on their disease severity.

Subjects with COPD, asthma and IPF usually face distinct challenges when intending to engage in physical activities. For instance, those with COPD often present airflow limitation, which leads to dyspnoea and exercise intolerance in the long term, making it difficult for them to keep active on a daily basis [10]. In a different way, asthmatic subjects may experience intermittent symptoms triggered by environmental factors [8], impacting their capacity to exercise and consequently reducing their PA levels. Subjects with IPF are characterized by fibrotic lung tissue that can also cause significant dyspnoea sensation and peripheral muscle fatigue, leading to their inactive characteristic in daily routine [5,11]. Understanding these disease-specific factors is vital for tailoring not only pulmonary rehabilitation and exercise programmes [37,38,39], but also PA interventions to meet individual patient’s needs. This could help to optimize PA levels across the different chronic lung conditions, ultimately improving quality of life and overall health outcomes of the different populations.

Disease severity emerged as a key factor influencing PA levels in the present study. On this topic, our findings underscore the importance of assessing PA patterns in patients with chronic lung diseases, especially in severe and very severe subjects. As seen in Table 2 and Figure 2, patients with severe and very severe IPF were the most inactive subjects of our sample. This may highlight the importance of patient-centred care [38] in managing their own PA patterns. Thus, education, individualized exercise treatments, PA counselling and self-management strategies [38,40] that contemplate varying disease severities and patients’ preferences seem to be essential components of care. Empowering chronic lung disease subjects to make informed decisions about their daily PA levels and actively participate in their care can lead to more positive outcomes, particularly when considering the most clinically debilitated individuals.

Although intuitive, the fact that lung function has different characteristics among the studied populations does not automatically mean that PA levels are also different among the three groups. In fact, it is well established by the scientific literature that variables of pulmonary function (such as FEV_1_ and FVC) correlate only modestly with PADL levels and do not show consistent effects on PA [3]. Since PADL is an important determinant of worse outcomes in subjects with lung diseases, it is of the utmost importance to increase PA levels in order to mitigate and counteract the deleterious effects of inactivity in these groups of subjects. However, to be able to do so, one should: (i) understand the varying levels of PA in subjects with different chronic lung conditions; (ii) compare and explore the possible differences in daily PA levels according to diseases’ severity; and (iii) understand that different populations might need different interventions to enhance PADL. Once these points are recognized, interventions using clear and specific strategies for each subgroup of subjects might be outlined to achieve a successful goal of improving PA.

Current physical activity guidelines [41] recommend adults and older adults with chronic conditions to engage in at least 150 min of moderate-intensity or at least 75 min of vigorous-intensity aerobic PA over the week for significant health benefits. Despite being more active than subjects with COPD and IPF, our results show that asthma subjects (on average) are still less active in daily routine and do not achieve the amount of PA recommended by international guidelines. This lower level of daily PA may be influenced by several factors in the different conditions [3,6,11]. In order to stimulate subjects with chronic lung diseases to achieve higher levels of daily PA, it seems important to consider the whole spectrum of an individual’s needs, taking into account, among other characteristics, the type and severity of their disease. Therefore, healthcare professionals and researchers must consider tailoring the interventions according to subjects’ abilities by setting realistic goals. Hence, strategies aiming at increasing PA levels should focus on: progressively enhancing PA by making subjects do small amounts of PADL; gradually increasing the duration, frequency and intensity of activities over time; and engaging in shorter bouts of activities, such as intervals/sessions of 10 min, which might be of importance to achieve these goals by the accumulation of PA throughout the week.

When considering the three investigated populations, taking into account the type, severity, pathophysiological mechanisms and some clinical particularities of each lung condition, we can suggest and speculate on possible ways to tackle physical inactivity in these subjects. As for the individuals with asthma, despite being more active than those with COPD and IPF, they could still enhance their activity levels by increasing the time spent in light activities and MVPA. Perhaps for individuals with COPD, they could engage in more MVPA by transforming the time they spent in light-intensity activities into moderate intensity, and those with a high degree of sedentarism could turn some of their sedentary time into light-intensity activities. Finally, for individuals with IPF, a possibility would be to first increase their exercise capacity, then decrease their time spent in sedentary activities by turning/translating them into more minutes of time spent in light-intensity physical activity. Once this stage is reached, these subjects could be induced to transform some of the time spent in light activities into MVPA. To achieve these goals, it seems important to consult with a health care professional or a physical activity specialist in order to define clear goals, not exceeding the physical and psychological demands that subjects with chronic lung conditions have. This ‘step-by-step’ approach seems particularly important when considering the most severe subjects.

While our study offers noteworthy insights into patterns of PADL among subjects with COPD, asthma and IPF, there are still areas that need further investigation in clinical research. For example, longitudinal studies with a follow-up period tracking changes in PA levels over time and assessing the long-term impact on disease progression are needed. In addition, the usefulness of tele-rehabilitation [38] and emerging technologies [42,43] in enhancing PA levels and promoting engagement could open new possibilities of treatments for improving care in these populations. Lastly, using a personalized approach considering patients’ need, mainly in subjects with severe and very severe IPF, might boost the likelihood of sustained PA engagement and improved health outcomes.

The present study has some limitations. First, it has a cross-sectional design and it is a secondary analysis of baseline data from three different projects. Still, data collected for the present analysis were standardized and each independent project used the same tests and devices to measure the outcomes, which made it possible to compare the varying chronic respiratory diseases. Nonetheless, future research should consider longitudinal approaches with a larger sample size to better investigate the dynamic nature of daily PA levels in these lung conditions, with a closer look into different diseases’ severity. Another possible limitation could be the number of subjects in the IPF group, which was smaller than both the COPD and asthmatic groups. However, this can be partly explained by the epidemiology of the disease, which is rarer than the other two populations [44]. On this topic, we chose specifically to not include all subjects with ILD (e.g., non-IPF) to not expose our results to sample heterogeneity. Climatic variations could have influenced the PA levels in the present study. However, all projects had a ‘rolling’ start, and therefore subjects were assessed throughout the whole year, reducing the chance of seasonal bias. Moreover, since the data collection period was relatively similar among the projects, the season when baseline data collection was conducted was fairly consistent across all three projects. Finally, most of the asthmatic subjects still work regularly (e.g., are not retired) and this could have influenced PA levels in this group of patients. However, we do believe that this is a specific characteristic of this population, which is younger than both COPD and IPF subjects. Although we did not correct for working status, we did correct our analysis for age and we truly believe it reflects the differences among these three chronic major respiratory diseases.

In conclusion, the present study highlights the significant differences in PA among subjects with COPD, asthma and IPF, emphasizing the influence of disease type and severity on activity levels. Our findings highlight that subjects with asthma are more active in their daily life than subjects with COPD and IPF. Furthermore, there are no differences in PA levels when comparing mild/moderate subjects in the different subpopulations. However, individuals with severe and very severe IPF are the most inactive ones. These disease-specific patterns of PA underscore the importance of tailoring interventions to address these discrepancies and help the unique challenges that each subgroup faces.

## Figures and Tables

**Table 1:** Characteristics of the studied subjects.

**Variables**	**Asthma (*n* = 47)**	**COPD (*n* = 48)**	**IPF (*n* = 24)**
Age, years	48 ± 15 ^a,b^	66 ± 9	63 ± 9
Sex, M/F	16/31	26/22	15/9
BMI, kg/m^2^	28 ± 6	29 ± 5	28 ± 5
FVC, %pred	86 ± 16 ^b^	80 ± 19	67 ± 19 ^a^
FEV_1_, %pred	73 ± 17 ^a^	51 ± 18	70 ± 19 ^a^
FEV_1_/FVC	70 ± 11 ^a,b^	51 ± 11	84 ± 6 ^a^
6MWT, meters	551 ± 95 ^a,b^	443 ± 92	440 ± 101
6MWT, %pred	98 ± 13 ^a,b^	84 ± 17	77 ± 24
QF_MVC, Kgf	21 ± 12 ^b^	24 ± 10 ^+^	36 ± 14 ^a^

Sex, M: male, F: female; BMI: body mass index; FVC: forced vital capacity; FEV_1_: forced expiratory volume in the first second; %pred: percentage of predicted values; 6MWT: distance walked in the six-minute walking test; QF_MVC: maximal voluntary contraction of the quadriceps femoris; Kgf: kilogram force. ^a^ *p* < 0.05 vs. COPD; ^b^ *p* < 0.05 vs. IPF. ^+^ *n* = 31.

**Table 2:** Characteristics of physical activity levels in subjects with severe/very severe chronic respiratory diseases, corrected for age, BMI and sex.

Variables	Asthma (*n* = 32)	COPD (*n* = 24)	IPF (*n* = 6)	*p*-Ancova
Sedentary, min/day	383 (354–412)	367 (333–400)	437 (372–502)	0.152
Light, min/day	269 (244–294) ^b^	232 (203–260)	148 (92–203) ^a^	**0.001**
MVPA, min/day	18 (13–24) ^a^	7 (1–13)	2 (−10–15) *	**0.027**
Steps/day, n/day	5643 (4750–6536) ^b^	4273 (3248–5298)	2324 (338–4309)	**0.011**

Values expressed as the mean (95%CI). Sedentary: mean time spent/day in sedentary activities (minutes/day). Light: mean time spent/day in light activities (minutes/day). MVPA: mean time spent/day in moderate-to-vigorous physical activity (minutes/day). Steps/day: mean number of steps/day. ^a^ *p* < 0.05 vs. COPD; ^b^ *p* < 0.05 vs. IPF; * near-significance vs. asthma. Bold values indicate statistically significant differences.

## Data Availability

The data presented in this study are available on request from the corresponding authors. The data are not publicly available due to sensitive information and ethical restriction.
